# Comparing Cyclic Fatigue Resistance and Free Recovery Transformation Temperature of NiTi Endodontic Single-File Systems Using a Novel Testing Setup

**DOI:** 10.3390/ma17030566

**Published:** 2024-01-25

**Authors:** Emad Youssef, Holger Jungbluth, Søren Jepsen, Manfred Gruener, Christoph Bourauel

**Affiliations:** 1 Department of Periodontology, Operative and Preventive Dentistry, University of Bonn, 53111 Bonn, Germany; 2 Department of Oral Technology, Faculty of Medicine, University of Bonn, 53111 Bonn, Germany

**Keywords:** bend and free recovery, cyclic fatigue, reciprocating endodontic instruments

## Abstract

The aim of this study was to assess the effect of body temperature (37 °C) on the cyclic fatigue resistance of three endodontic single-file systems using a new testing setup. One Shape^®^ new generation (OS), WaveOne™ (WO) and WaveOne^®^ GOLD (WOG), which are made from different NiTi alloys and operated in different motions (rotation/reciprocation), were evaluated. The study design included four groups. Each group comprised 30 files, 10 files of each of the three file systems, tested at 20 ± 2 °C (group 1 and 3) and at 37 ± 1 °C (group 2 and 4). All files were tested in a custom-made metal block with artificial canals of 60° angle, and a 5 mm and 3 mm radius of curvature, respectively. A heating element was attached to replicate a temperature of 37 °C. Files were introduced 18 mm into the canals and operated until failure. Transformation temperatures of five samples of each of the tested file systems were determined via the bend and free recovery (BFR) method. With the exception of WOG in canals with a 3 mm radius of curvature (*p* = 0.075), all the tested file systems showed statistically significantly less time needed to fracture when operated at 37 ± 1 °C compared to at 20 ± 2 °C in canals with a 5 mm and 3 mm radius of curvature using Mann–Whitney U test (*p* < 0.05). All file systems showed transformation temperatures below the body temperature. We concluded that body temperature directly affects the cyclic fatigue resistance of all tested file systems. Bend and free recovery can be suitable for the determination of austenite finish temperatures (A_f_) of endodontic instruments as it allows testing a longer portion of the instrument.

## 1. Introduction

Root canal instrumentation requires the use of highly flexible instruments that can easily bend to clean and shape curved root canals while respecting the original canal anatomy. Due to its low modulus of elasticity, nickel-titanium (NiTi) alloy gained interest in being used in fabricating root canal instruments in the last three decades.

In 1988, Walia et al. were the first to report that K-files #15 manufactured from NiTi alloy showed two to three times more elastic flexibility in bending and in torsion in addition to higher torsional fracture resistance compared to stainless steel counterparts that had the same size and the same manufacturing process [1].

In 1992, the first generation of rotary 0.02 taper NiTi files came to the market and its introduction revolutionized the practice of endodontics and changed the way of mechanical canal instrumentation [2]. Engine-driven rotary canal instrumentation allowed faster canal preparation and minimized procedural errors associated with hand instrumentation, which was advantageous for both experienced and inexperienced operators [3].

Despite its many advantages, a major concern with using engine-driven NiTi instruments was their liability to fracture [4,5]. Separation of engine-driven NiTi instruments can occur due to either torsional fracture (shear failure) or cyclic fatigue (flexural fatigue) or a combination of both [4,6]. Many NiTi file systems were developed to reduce the incidence of instrument separation inside root canals. The major changes made were in terms of instrument design, type of cutting motion inside the root canal (full rotation and reciprocation), alloy of the instrument and method of instrument fabrication (heat treatment and electrical discharge machining).

In order to better understand the reasons for instrument separation, many in vitro studies were conducted comparing the behavior of different file systems in standardized artificial canals with different radii and angles of curvature [7,8,9,10,11]. Not until recently, all of the available in vitro studies on cyclic fatigue were conducted at room temperature. NiTi is considered to be a shape memory alloy that exhibits pseudoelasticity (super-elasticity) and the shape memory effect [12]. The shape memory effect refers to the ability of a material to restore its original shape after heating. This indicates that NiTi can be sensitive to temperature changes and shows different behaviors in different temperatures due to different specific heat capacities of different material phases [13].

Most of the engine-driven root canal instruments are made of 55 Nitinol alloy [≈56% (wt) nickel and 44% (wt) titanium] and characteristically have two phases, an austenitic phase where the crystal structure of the alloy is a stable, body-centered cubic lattice, and a martensitic phase, with the crystal structure of a closely packed hexagonal lattice [14]. Temperature or stress-induced change between these two phases can occur, resulting in the previously mentioned characteristics termed shape memory and super-elasticity. 

When an NiTi instrument is used to clean and shape a curved root canal in a patient’s tooth, it is subjected to two different hysteresis, stress-induced martensitic transformation (due to canal curvature) and, simultaneously, heat-induced austenitic transformation (due to body temperature). Therefore, in our study we wanted to reproduce these conditions of files operated at body temperature in comparison to room temperature in curved canals.

In this study, we compared the cyclic fatigue resistance of three single-file systems, One Shape^®^ new generation (OS), WaveOne™ (WO), WaveOne^®^ GOLD (WOG), which have the same tip diameter (iso #25), made from different alloys and operated in different motions (rotation/reciprocation). Bend and free recovery testing was then used to assess the transformation temperature of the three file systems included in this study.

The null hypothesis is that there is no difference in cyclic fatigue characteristics of NiTi instruments, when driven at room temperature or at body temperature. 

## 2. Materials and Methods

**Figure 1:**
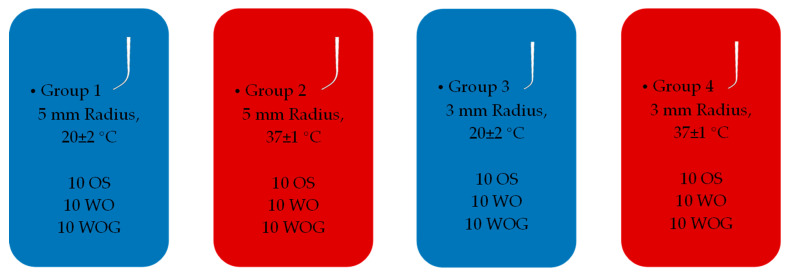
Groups distribution.

In this study, a total of 120 files divided into 4 groups were included. Each group comprised 30 files, 10 files from each of the tested three file systems: One Shape^®^ new generation (OS) (MICRO-MEGA, Besançon, France), WaveOne™ Primary (WO) (Dentsply Maillefer, Ballaigues, Switzerland) and WaveOne^®^ GOLD primary (WOG) (Dentsply Maillefer, Ballaigues, Switzerland). Figure 1 shows the groups distribution. 

In Group 1 (G1) and Group 3 (G3), the files were tested in a metal block with artificial canals of a 5 mm and 3 mm radius of curvature at room temperature 20 ± 2 °C. In Group 2 (G2) and Group 4 (G4), the files were tested in artificial canals with a 5 mm and 3 mm radius of curvature at simulation of body temperature of 37 ± 1 °C. In all groups, the artificial canal angle of curvature was kept the same at 60° as determined by Pruett et al. [15]. 

### 2.1. Cyclic Fatigue Testing

#### 2.1.1. Testing Setup

**Figure 2:**
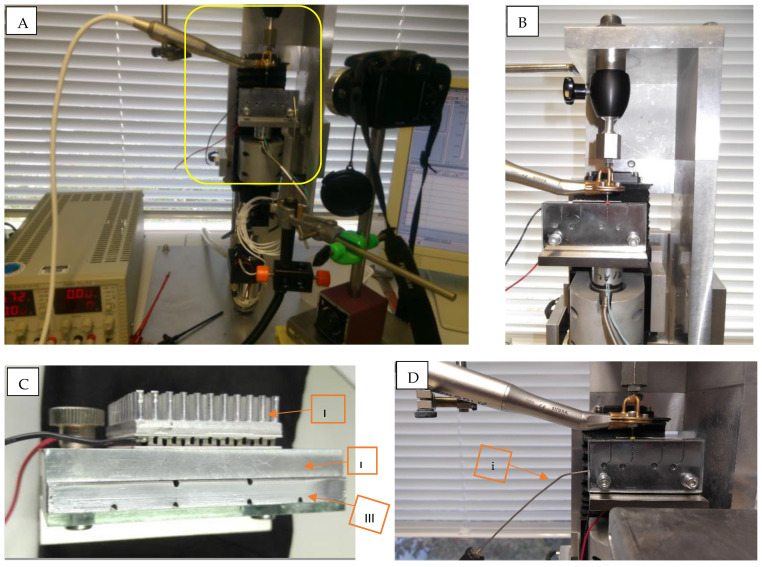
(**A**) Testing setup. (**B**) Testing block attached to L-shaped block holder which is kept in place in testing machine by means of electromagnet. Handpiece with tested file is mounted. (**C**) Peltier element (I) attached to L-shaped holder (II) and testing block (III) is fixed in place with a screw. (**D**) Thermometer metal probe (i) placed in a hole drilled in the testing block to measure core temperature of the block.

A self-developed mini universal testing machine was used (Figure 2). A handpiece holder was fixed to the upper end of the machine while the stainless steel block (manufacturing method is described in Appendix A) was attached to the lower part (moving part). The stainless steel block was fixed to a stainless steel holder that assured correct and reproducible positioning. The holder thickness was 8 mm and was equipped with a Peltier element (semiconductor-heating element) attached to it as shown in Figure 2C. The Peltier element was connected to a power control box for adjusting the current and voltage needed to generate a temperature of 37 ± 1 °C in the stainless steel block. The holder was fixed to the lower part (moving part) of the testing machine by means of an electromagnet connected to a force detecting sensor.

Using the metal holder allowed a uniform heat transfer to the stainless steel testing block and easy removal of the block, for retrieval of the separated instrument fragments, without changing the position relation between the handpiece and the block holder. Keeping a fixed relation between the handpiece holder and block holder allowed the reproducibility of file position in the canals after removing the separated fragment. The force sensor connected to the magnet holding the block holder ensured the correct insertion angle each time a new file was used. The force sensor readings showed that the files were introduced in the canal freely (at 0° inclination) until first contact with the canal curvature as inclined insertion angle can affect the cyclic fatigue of tested instruments [16].

The universal testing machine was connected to a computer where the force sensor readings can be monitored, and through a self-developed controlling program, the introduction of the files in the canals was controlled. The files were introduced 18 mm inside the canals after lubricating the canals with non-CFC propellant (KaVo Spray™, KaVo Dental GmbH, Biberach an der Riss, Germany). A camera setup (S5700 Fujifilm Group, Tokyo, Japan) was placed directly in front of the glass cover and an FHD video recording was started, and was used later for confirming the time to fracture. A metal probe thermometer (AZ 8856, Taichung, Taiwan) was placed in a 7 mm deep hole drilled on the side of the stainless steel testing block for continuous monitoring of temperature during testing (Figure 2D). The 6:1 reduction handpiece (Sirona Dental Systems GmbH, Bensheim, Germany) was connected to an endomotor (Reciproc Gold, VDW, Munich, Germany) and the operating mode was chosen according to the manufacturer’s instructions. For WOG and WO, the mode “Waveone All” was chosen, where both the instruments were operated in a partial reciprocation movement with a forward angle of rotation of 150° and a reverse angle of 30°. For OS, the speed was set to 350 rpm and the torque was set to 2.5 N.cm.

#### 2.1.2. Testing Procedure

The rotation/reciprocation of files in artificial canals was controlled through the foot pedal and stopwatch. Operation was ceased upon visual or auditory separation detection. The retrieval process included lowering the block holder with the block, unscrewing the stainless steel block and storing the separated fragment along with the rest of the tested file. After attaching a new file to the handpiece and fixing the block to its holder, the block and its holder were moved up as an assembly until the new file was introduced 18 mm in the artificial canal to start a new test cycle. Tests of G1 and G3 were conducted in an air-conditioned room with a fixed temperature set to prevent temperature changes. The setup was left overnight in the room and temperature was recorded by the thermometer attached to the testing block during each test. The recorded temperatures were found to be in the range of 20 ± 2 °C. For G2 and G4, the temperature was continuously monitored and recorded during the testing procedure and the current intensity was adjusted (if needed) simultaneously to maintain the real-time temperature in the range of 37 ± 1 °C. 

### 2.2. Assessment of Transformation Temperature of NiTi Using Bend and Free Recovery Test

**Figure 3:**
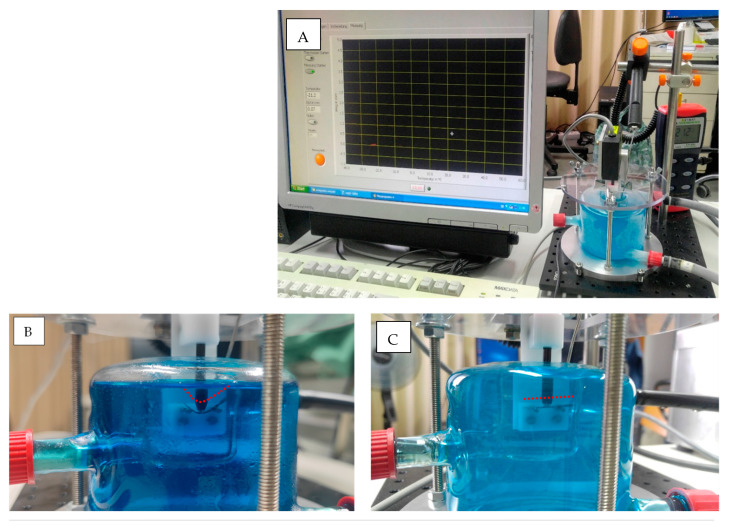
(**A**) Bend and free recovery testing setup showing the double chamber glass, laser sensor, thermometer and the connected computer for real-time plotting of displacement/temperature changes. (**B**) Closer look at the bent sample. (**C**) Straightening of the sample after reaching active A_f_ temperature. At both (**B**,**C**), a red dotted line parallel to the sample is drawn for illustration.

A self-developed bend and free recovery (BFR) testing machine was used (Figure 3). The machine was built following the ASTM international standard “Standard test method for determination of transformation temperature of Nickel-Titanium shape memory alloys by bend and free recovery” (Designation: ASTM F2082-03) [17]. The machine consists of a fluid pump with a thermostat, double-chambered glass container, displacement laser sensor, sample holder, carbon fiber rod, thermometer and a computer. 

Three groups (*n* = 5) representing the three used file systems were included. Samples of 20 mm (from the tip) were prepared by cutting the shaft of each file with a diamond disk. Laser sensor, thermometer and thermostat were started, all connections to the computer were checked and the measuring software was started on the computer. On the double-pin sample holder, which is placed inside the smaller chamber of the glass container, a 20 mm file sample was placed. The carbon rod was then seated on the straight sample nearly in the middle of the sample (Figure 3) and the thermometer was placed inside the smaller chamber not touching the sample.

Four liters of cooling liquid, pre-cooled at −20 °C for 24 h and at −80 °C for 3 h, were then poured into the pump and the pump was started. When the liquid filled the bigger chamber in the glass container, the cooling liquid was filled carefully inside the smaller chamber using a 20 mL syringe. The carbon rod was then pressed 2–4 mm down while resting on the cooled sample (martensite phase), which resulted in curving the sample with max. point of curvature near the mid-length (Figure 3B). The laser sensor was then focused on the carbon rod end and the zero position was recorded. When the coldest temperature was recorded, the measurement cycle was started. The pump was previously set to heat the liquid that was pumped to the bigger chamber of the glass container. The liquid in the smaller chamber was heated by heat conduction and the temperature was recorded continuously. 

The rise in temperature of the liquid in contact with the sample resulted in a phase transformation of the NiTi and caused the sample to straighten back to its original straight form. When the sample straightened back, it caused the freely moving carbon rod resting on the sample to move upwards and the laser sensor recorded this displacement. The recorded displacement with the continuously recorded temperature data was used to plot a graph that shows the transformation temperature due to free recovery of the tested sample.

### 2.3. Scanning Electron Microscopy

The fractured segments underwent analysis with a scanning electron microscope (XL30 SEM, Philips, Eindhoven, The Netherlands) to validate that the fracture occurred as a result of cyclic fatigue, displaying the characteristic cyclic fatigue fracture pattern.

### 2.4. Statistical Analyses

The assumption of normal distribution of data was tested using the Shapiro–Wilk test. The time to fracture data for each file system tested at two different ambient temperatures or in canals with two different radii were analyzed via the Mann–Whitney U test, while the analysis of time to fracture between different file systems within the same group was done using Mood’s median test. IBM SPSS Statistics for Windows (Version 23.0., Armonk, NY, USA: IBM Corp) was used for all statistical analyses.

## 3. Results

### 3.1. Cyclic Fatigue Resistance

**Figure 4:**
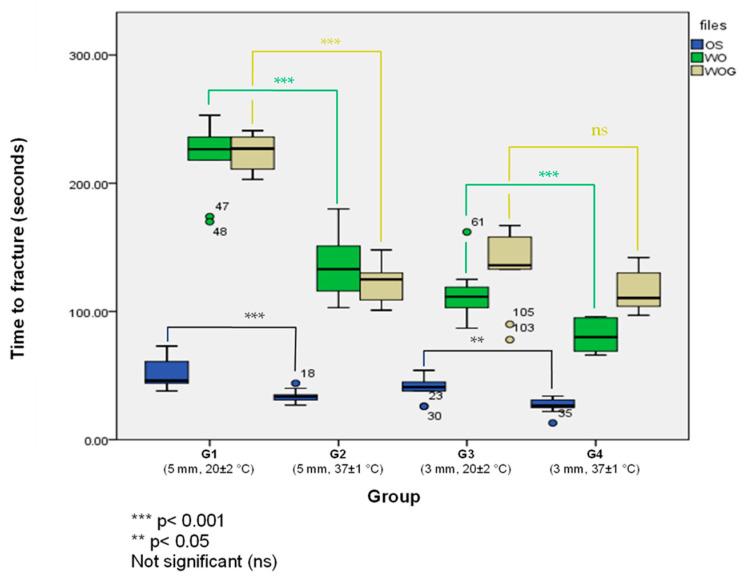
Mean time to fracture by groups. Group 1 (G1): 5 mm 20 ± 2 °C, Group 2 (G2): 5 mm 37 ± 1 °C, Group 3 (G3): 3 mm 20 ± 2 °C, Group 4 (G4): 3 mm 37 ± 1 °C.

Table 1 shows the mean time to fracture (in seconds) and standard deviation for One Shape^®^ new generation (OS), WaveOne™ (WO) and WaveOne^®^ GOLD (WOG) at room temperature (20 ± 2 °C) and body temperature (37 ± 1 °C). When tested in artificial canals with a 5 mm radius of curvature, all tested file systems showed statistically significantly less time to fracture at body temperature compared to room temperature (*p* < 0.05). In canals with a 3 mm radius, both WO and OS showed the same results, while time to fracture at body temperature was statistically significant lower compared to when tested at room temperature (*p* < 0.05) (Figure 4). For WOG, the statistical difference was insignificant at the 95% confidence level (*p* > 0.05). At both, room temperature (20 ± 2 °C) and body temperature (37 ± 1 °C), both of OS and WO, showed statistically significantly less time to fracture when tested in artificial canals with a 3 mm radius compared to artificial canals with a 5 mm radius of curvature (*p* < 0.05). For WOG, the time needed to fracture in 5 mm canals was significantly longer than in 3 mm canals at room temperature (*p* < 0.001), but when tested at body temperature, WOG instruments showed no difference in time to fracture between 5 mm and 3 mm canals (*p* = 0.353).

Comparing the three file systems within the same group showed that OS (Rotating motion) had statistically significantly less time to fracture in all tested conditions compared to the two other file systems WO and WOG (reciprocating motion) (*p* < 0.001). Comparing the time to fracture of both reciprocation systems, WO and WOG, showed that in canals with a 5 mm radius of curvature, no statistical significance was detected at both testing temperatures (*p* > 0.1). When tested in canals with a 3 mm radius of curvature, WOG had statistically significantly longer time to fracture compared to WO at both testing temperatures (*p* < 0.007). 

### 3.2. Transformation Temperature by Bend and Free Recovery

**Figure 5:**
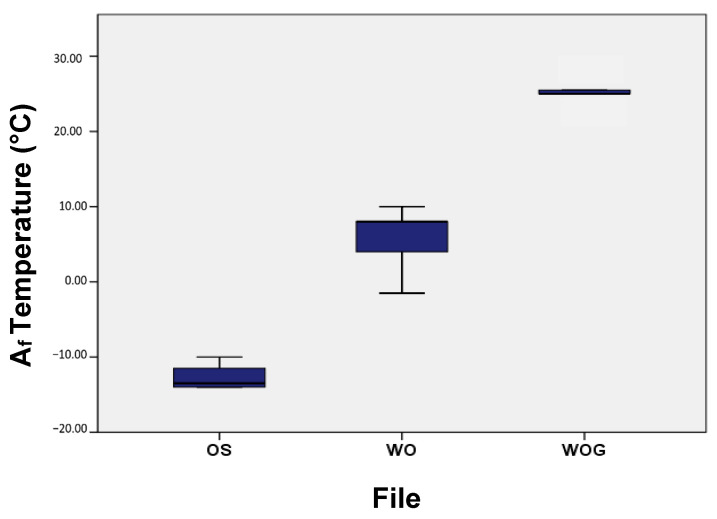
Mean active austenite finish (A_f_) temperatures as determined by bend and free recovery test.

**Figure 6:**
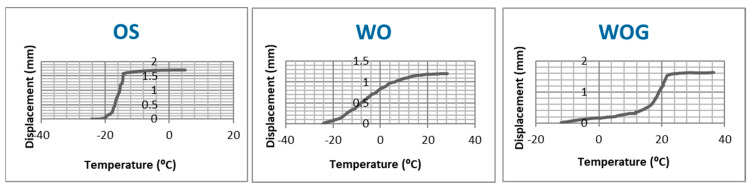
Active A_f_ Temperature of OS, WO and WOG as determined by bend and free recovery testing.

Table 2 shows the mean and standard deviation of active austenite start (A_s_) and active austenite finish (A_f_) temperatures as determined by the bend and free recovery test. WOG showed the highest A_s_ and A_f_ temperatures, while OS showed the lowest (Figure 5). All tested samples had an Af temperature below body temperature (37 ± 1 °C) (Figure 6).

### 3.3. Scanning Electron Microscopy

**Figure 7:**
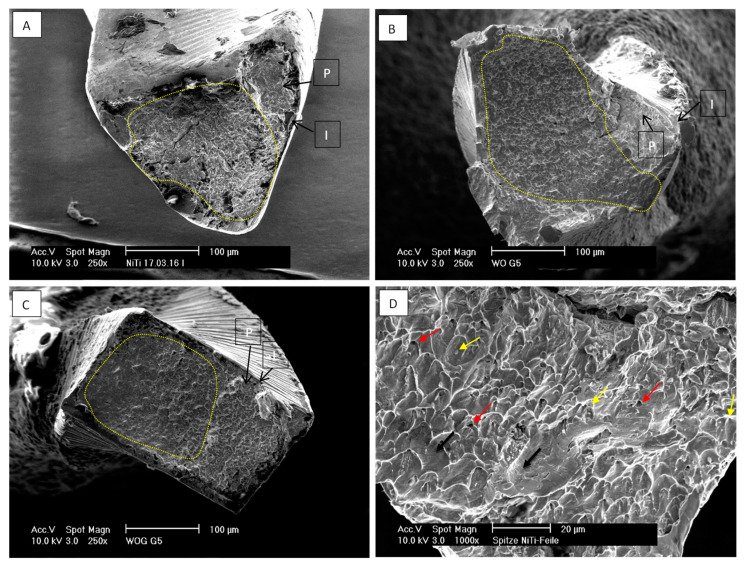
Scan electron microscope images of OS (**A**), WO (**B**) and WOG (**C**) where areas of crack initiation (I) and propagation (P) are identifiable. Area marked with dotted line shows catastrophic ductile failure. (**D**) Red arrows: micro-voids, yellow arrows: Inclusions/cones and black arrows: fatigue striations.

Analysis of the cross-section of fractured surfaces using scanning electron microscopy (XL30 SEM, Philips, Eindhoven, The Netherlands) showed characteristic fatigue fracture patterns. As shown in Figure 7, areas of crack initiation (I), crack propagation (P) and areas of catastrophic fracture with ductile failure (dotted line) that showed prominent dimple defects are identifiable. Micro-voids (Figure 7 red arrows) and inclusions (cones) (Figure 7 yellow arrows) of different shapes and sizes were also observed distributed among the fracture surface with higher magnification. Fatigue striations (black arrows) could also be detected at higher magnification.

## 4. Discussion

New advancements are introduced continuously to present new engine-driven instruments with improved properties that can provide better performance in various clinical situations. One of these advancements was the introduction of partial reciprocating motion of endodontic files with rotation effect, where the instrument rotates in the canal with an angle of rotation in the forward cutting direction greater than the angle of rotation in the non-cutting opposite direction [18]. The instrument completes one turn after a certain number of reciprocating cycles [19]. Both file systems that were reciprocating (WO and WOG) showed significantly longer time to fracture in all tested groups when compared to OS, which was operated in full rotation. This confirms the findings of previous studies that showed that reciprocating instruments needed significantly longer time to failure compared to the full rotation instruments [20,21,22,23,24,25]. These results can be attributed to the fact that reciprocating instruments need more time to complete the same number of cycles as full rotation instruments or they are operated at a lower rpm.

When NiTi instruments are introduced into a curved canal, stress exerted from the curvature causes a stress-induced martensite transformation within the instrument. Thermal-induced phase transformation of NiTi is a known feature of these alloys, where a martensite to austenite transformation happens upon heating [14]. In 2016, body temperature was recognized as a factor that can alter the cyclic fatigue resistance of endodontic file systems [26]. Our results showed that when tested in moderately curved canals with a 5 mm radius of curvature, all tested file systems needed significantly less time to fracture at body temperature compared to room temperature (*p* < 0.05). In canals with a 3 mm radius, both WO and OS showed the same results, where time to fracture at body temperature was statistically significant lower compared to when tested at room temperature (*p* < 0.05). These findings confirm the results of previous studies reporting that environmental temperature has an influence on the cyclic fatigue resistance of NiTi-shaping instruments [26,27,28,29,30,31]. For WOG, the statistical difference was insignificant at 95% confidence (*p* > 0.05). Although no statistically significant difference was found for WOG when tested in canals with a 3 mm radius, the mean time to fracture at body temperature (115 ± 15 s) was still shorter than at room temperature (134 ± 30 s). This finding could be attributed to the sample size (*n* = 10) and a statistical significance might be detected with a larger sample size.

In 1997, Pruett et al. reported that there was an inverse relation between the radius of canal curvature and the cyclic fatigue resistance of NiTi instruments [15]. In our study, canals with 3 mm and 5 mm radii of curvature were used to represent canals with severe and moderate curvatures [32] that can be difficult to manage clinically. Our results showed that all tested file systems needed statistically significantly less time to fracture when tested in artificial canals with a 3 mm radius of curvature compared to artificial canals with a 5 mm radius at room temperature (20 ± 2 °C). This is consistent with previous findings in the literature [15,16,33,34]. At body temperature (37 ± 1 °C), both OS and WO showed the same findings, where time to fracture was significantly lower in 3 mm than in 5 mm canals (*p* < 0.005); however, WOG showed different results, where no significant difference could be observed between the time to fracture in 3 mm and 5 mm canals.

Resistance to cyclic fatigue can be influenced by various factors including the instrument design (taper, cross-section, central core mass, etc.) and alloy of the instrument [35]. The tested file systems in our study have the same tip diameter but they have different cross-sectional geometries. The OS cross-section at the apical portion is a modified triangle with a symmetrical radius and three cutting edges, the middle of the instrument has a transition to two cutting edges and the coronal portion has an S-shaped cross-section with two cutting edges. WO has a convex triangular cross-section coronally and a concave triangle at the apical portion. WOG has a parallelogram-shaped cross-section with two cutting edges in contact with the canal wall, alternating with an off-centered cross-section where only one cutting edge is in contact with the canal wall [36]. OS has a constant taper of 6% along the cutting part of the instrument, while both WO and WOG are designed to have a constant taper of 8% and 7%, respectively, for the most apical 3 mm. Then both instruments have a variable/regressive taper until the end of their cutting portion. The used instruments OS, WO and WOG were made of conventional super-elastic NiTi, M-wire and G-wire, respectively. When we compared the time to fracture of WO and WOG tested at the same group, we observed no significant difference in moderately curved canals (5 mm) (*p* > 0.05) but in severely curved canals (3 mm), WOG needed significantly longer time to fracture than WO (*p* < 0.05). This shows that WOG had a superior performance when compared to WO in severely curved canals.

For testing the cyclic fatigue of engine-driven file systems, many studies used a setup where the files were immersed in a water or saline bath that was heated to body temperature (37 ± 1 °C) [37,38,39,40]. Yum et al. [41] explained the galvanic corrosion that happened to Protaper Universal (PT) (Dentsply Maillefer, Ballaigues, Switzerland) instruments. They reported that EDX micro analysis of PT instruments revealed that the cutting and non-cutting sections of the instruments were made of NiTi, while the shank was made of gold-plated Brass and when in a galvanic cell, the NiTi acts as the anode and the shank acts as the cathode. Although the effect of galvanic corrosion can be insignificant due to the short testing duration (minutes), we used a new setup where a Peltier heating element attached to metal holder transferred the heat to the testing block. The metal probe thermometer placed in a hole drilled directly in the testing block served for accurate real-time monitoring of the core temperature in the testing block. 

The bend and free recovery (BFR) test and differential scanning calorimetry (DSC) are two methods used for measuring the transformation temperatures of nickel-titanium (NiTi) shape memory alloys [42]. The DSC monitors the heat flow during both cooling and heating, whereas BFR records the deflection recovery only during heating [42]. The key transformation temperatures austenite start (A_s_) and austenite finish (A_f_) can be determined using either method. BFR can be performed directly on finished products and simulates the actual conditions that a product will experience in use, providing a more realistic evaluation of its performance. The bend and free recovery (BFR) method was used to determine the active austenite finish temperature (A_f_) of the instruments. Using this method allowed us to test a 20 mm fragment of the instrument including the whole cutting section of the instrument (16 mm), unlike the case when differential scanning calorimetry (DSC) was used, where a small sample (usually 5 mm from the tip portion of the instrument) was used to determine the martensite–austenite transformation temperatures. All of the tested file systems showed A_f_ below body temperature (37 ± 1 °C), indicating that they are predominantly in the austenitic phase at body temperature. Scott et al. reported different results for WO and WOG using DSC [43]. Differences between transformation temperatures determined by BFR and DSC were previously reported [44]. The reasons for these differences are not well understood but it was observed that DSC measures the transformation temperatures in the absence of any external stress, and the transformation temperatures may be affected by residual stress, thermal impedance of the sample geometry and surface condition [45]. Also in DSC, cutting and stacking an acceptable sample weight can affect the stress state and the thermal impedance of the wire in the test cell [45]. 

WOG showed the highest A_f_ temperature (25 ± 2 °C) among the tested samples. Alapati et al. reported that M-wire, from which WO instruments are made, shows the three NiTi crystalline phases, martensite, R-phase and austenite [46]. WO showed a wider range between A_s_ and A_f_, indicating the possibility that the phase transformation within the WO instrument is a multi-stage transformation [47]. WOG had an A_f_ temperature (25 ± 2 °C) higher than that of the used room temperature (20 ± 2 °C) and was expected to outlast WO instruments when operated at room temperature; however, our results showed that this happened only in severely curved canals with a 3 mm radius. In 5 mm canals, no significant difference between WO and WOG was observed in terms of time needed to fracture, which is different from previously reported results [48]. When comparing these two file systems, it should be taken in consideration that they have a different taper, different cross-section designs and are made from different alloys, so these factors can affect their cyclic fatigue resistance [35].

## 5. Conclusions

Body temperature directly affects the cyclic fatigue resistance of all tested single-file systems and is considered as a factor altering the cyclic fatigue resistance of NiTi endodontic instruments. Single-file systems operated in reciprocation are more resistant to cyclic fatigue failure than those operated in full rotation. The active austenite transformation temperature determined by BFR allows for testing samples in a pre-bent or pre-stressed state, effectively imitating the stresses exerted on endodontic instruments in curved root canals. This emphasizes the ability of BFR to mimic real-world conditions, making it a more relevant and practical method for evaluating the transformation temperature of endodontic instruments in their final product form.

## Figures and Tables

**Table 1:** Mean time to fracture (in seconds).

	Mean Time to Fracture (Seconds)				*p*-Value	
	Group 1 (G1) (5 mm, Room temperature 20 ± 2 °C)	Group 2 (G2) (5 mm, Body temperature 37 ± 1 °C)	Group 3 (G3) (3 mm, Room temperature 20 ± 2 °C)	Group 4 (G4) (3 mm, Body temperature 37 ± 1 °C)	G1–G2	G3–G4
One Shape^®^ new generation (OS)	52 ± 12	34 ± 5	40 ± 9	26 ± 6	<0.001	0.002
WaveOne™ (WO)	221 ± 28	135 ± 25	114 ± 21	81 ± 12	<0.001	<0.001
WaveOne^®^ GOLD (WOG)	224 ± 14	122 ± 15	134 ± 30	115 ± 15	<0.001	0.075

**Table 2:** Mean active austenite start (A_s_) and mean active austenite finish (A_f_) temperatures as determined by bend and free recovery test.

	A_s_ (°C)	A_f_ (°C)
OS		
Mean	−17	−12
SD	3	2
WO		
Mean	−14	6
SD	3	5
WOG		
Mean	21	25
SD	3	2

## Data Availability

Data are contained within the article.

## Appendix A

Testing block manufacturing: A custom-made block made from stainless steel for medical use (A316) was used for this study, where the artificial canals were engraved using spark erosion processing. The depth of all canals was greater than the shaft diameter of all the files used. All canals were manufactured as described by Plotino et al., 2010 [49], with a relief of 0.1 mm greater than the tested files diameter to ensure the free rotation/reciprocation of the files inside the canals and to exclude fracture of the files due to torsional overload. The metal block had a glass cover fixed with two screws that allowed visualization of the files while testing to record the time needed to fracture and prevented file slippage. The inner surface of the artificial canals had a chrome-hardening layer with a thickness of 20 µm to avoid any dimensional changes in the canals due to friction and wear.
